# Miniaturization of Ocean Sensors Based on Optofluidic Technology: A Review

**DOI:** 10.3390/s25216591

**Published:** 2025-10-26

**Authors:** Wennan Zhu, Kai Sun, Weicheng Cui

**Affiliations:** 1 School of Engineering, Westlake University, Hangzhou 310030, China; zhuwennan@westlake.edu.cn (W.Z.); cuiweicheng@westlake.edu.cn (W.C.); 2 Key Laboratory of Coastal Environment and Resources of Zhejiang Province, School of Engineering, Westlake University, Hangzhou 310030, China; 3 Institute of Advanced Technology, Westlake Institute for Advanced Study, Hangzhou 310024, China; 4 Department of Electronic and Information Engineering, School of Engineering, Westlake University, Hangzhou 310030, China; 5 Zhejiang Key Laboratory of 3D Micro/Nano Fabrication and Characterization, Westlake Institute for Optoelectronics, Fuyang, Hangzhou 311421, China

**Keywords:** ocean sensors, optofluidic, micro robots, instruments miniaturization

## Abstract

The miniaturization of ocean parameter monitors is critical for environmental monitoring and oceanographic research. In recent years, rapid developments in data processing, artificial intelligence, micro-nano manufacturing and advanced materials have significantly improved sensing accuracy while reducing device size. The detection of key ocean parameters such as temperature, salinity, pressure, dissolved oxygen (DO), pH, nutrients, chlorophyll and so on is facilitated by these innovations. Among these emerging technologies, microfluidics and optofluidics have attracted large attention in the fields of biomedicine and environmental monitoring. These platforms have the advantages of high sensitivity, low power consumption and easy integration. Real-time and on-site monitoring can be achieved by them. Optofluidic technology shows great prospects for ocean sensing applications. Recent advances in optofluidic ocean sensors for the measurement of chemical parameters and their future potential are highlighted in this review. Ultimately, it presents the key role of optofluidic systems in realizing compact high-performance ocean parameter sensors. This plays an important role in paving the way for their integration into micro robots and the fourth generation of submersibles based on live fish.

## 1. Introduction

About 71% of the Earth’s surface is covered by oceans. This vast space makes the ocean the largest ecosystem on our planet. However, environmental changes such as ocean warming, acidification and pollution increasingly disrupt marine ecosystems and marine life activities. Meanwhile, human health is posed risks through contaminated seafood [1,2]. Continuous monitoring of key ocean parameters, such as dissolved oxygen, pH, nutrients, and chlorophyll and so on, is therefore crucial for understanding and solving these effects.

The 19th century is considered as a turning point in ocean science, where the first deep-sea survey was conducted. The HMS Challenger expedition (1872–1876) became the first global deep-sea survey. It collected various biological samples. Temperature and salinity profiles at various depths were also recorded in this period. These data laid the foundation for modern oceanography [3]. The advent of steam-powered vessels, deep-sea dredging tools and CTD (Conductivity, Temperature, Depth) profilers is also seen in this era [4].

Throughout the 20th century, ocean detection technologies advanced rapidly. And the monitors increasingly became complex and accurate. Sonar technology was first employed to explore underwater topography in the 1920s. This marked a significant leap in marine exploration [5]. By the 1950s, scientists were utilizing sonar systems to systematically map ocean ridges and deep-sea trenches. Our understanding of seafloor structures was greatly enhanced [6]. The advent of satellite remote sensing brought another transformative shift. In 1978, NASA launched the world’s first satellite, Seasat, to observe oceanic conditions from space. It was capable of measuring sea surface temperature, wind speed, and wave height. This innovation set the stage for a new era of ocean monitoring [7]. In the 1990s, the TOPEX/Poseidon satellite further pushed the boundaries of remote sensing. Its highly accurate data on sea surface height enabled scientists to detect and track sea-level changes with unprecedented precision [8]. A major breakthrough occurred in 1999 with the launch of the Argo program. Thousands of autonomous profiling floats across the globe were deployed in this plan. These buoys continuously collect real-time data on temperature, salinity, and ocean currents. It significantly enhanced spatial and temporal data coverage [9].

After the 21st century, ocean monitoring has become more integrated and globalized. The Global Ocean Observing System (GOOS) now combines data from a wide range of platforms, including satellites, buoys, autonomous underwater vehicles (AUVs) and so on. This action provided comprehensive, worldwide coverage [10]. Additionally, artificial intelligence and machine learning techniques are increasingly applied to analyze complex oceanographic datasets. It provided deeper insights and improved predictive capabilities.

Traditional oceanographic instruments are often bulky and expensive. These features significantly limit their deployment in remote or resource-constrained regions, such as the Arctic Ocean and deep-sea environments. Modern satellite-based ocean sensors provide large-scale coverage. But some limitations, like low spatial and temporal resolution cloud interference, cannot be ignored as they reduce accuracy and applicability in highly dynamic marine environments [11,12].

To overcome these limitations, researchers and engineers are becoming focused on the miniaturization of marine sensors. This shift has been further driven by two emerging technological trends. One is the development of micro robots [13,14,15], and the other is the development of the fourth generation of submersibles based on live fish [16].

Optofluidic technology is considered as a promising approach for the miniaturizing ocean sensors. Its origins can be traced back to the 1980s, where it emerged from the convergence of microfluidics and optical technologies. During this period, the development of microelectromechanical systems (MEMS) and early lab-on-chip (LOC) devices enabled precise manipulation of microscale fluid volumes. This established the foundation of microfluidics [17]. Simultaneously, the invention of optical tweezers by Arthur Ashkin in 1986 demonstrated the ability to manipulate microscopic particles using laser beams. It paved the way for light-based control in fluidic environments [18]. These pioneering efforts promoted the development of optofluidic technology.

The term “optofluidic” was formally introduced in 2003 by Holger Schmidt et al., and was further popularized by a seminal publication from Psaltis et al. in 2006 [19]. Psaltis et al. defined this novel field as the integration of optics and microfluidics [19]. In this period, innovations included liquid-core waveguides for dynamic light guiding using fluids, and tunable liquid lenses that adjusted focal length through electrowetting mechanisms [20,21]. By the late 2000s, optofluidic sensors utilizing surface plasmon resonance (SPR) enabled highly sensitive detection of biomolecules [22]. Meanwhile, the development of optofluidic dye lasers and photonic crystal structures expanded capabilities to include compact light sources and single-molecule detection [23,24].

Since 2010, the field of optofluidic has experienced rapid growth, particularly in applications in biomedicine and environmental monitoring [25,26,27,28,29,30]. In biomedicine, optofluidic platforms offer high sensitivity, compact design and non-invasive detection capabilities. These advantages enhance processes such as drug screening, diagnostics, and cell analysis. The late 2010s saw the integration of optofluidic systems with visible light communication (LiFi). High-speed, secure data transmission for real-time sensing can be achieved by LiFi [31]. In environmental monitoring, the combination of optofluidic systems with machine learning algorithms has improved the real-time detection of pollutants. It also helps the accuracy of data interpretation and intelligent decision-making [32,33]. Furthermore, rapid prototyping of complex optofluidic devices is enabled by advances in 3D printing and novel fabrication techniques. This facilitates the development of customized solutions for biosensors and environmental monitors [34,35,36]. The introduction of low-cost and easily processable materials has further broadened the accessibility and scalability of optofluidic systems.

The continued development of microfluidics and optofluidic has attracted considerable attention due to their potential as building blocks for compact, high-performance sensing systems. These technologies can integrate fluid handling and optical detection into a single microchip, enabling in situ analysis of chemical and biological parameters with low reagent volumes, often in the range of nanoliters to microliters, and power consumption as low as 1–2 W for portable devices [37,38,39]. Optofluidics is an extension of microfluidics that integrates optical components into fluidic systems to enable advanced light-based sensing, analysis and measurement. This review systematically explores the miniaturization of ocean sensors through the lens of optofluidic technologies, summarizing detection principles and representative LOC/optofluidic implementations for key chemical parameters in marine environments.

This paper provides an overview of current designs of miniaturized marine sensors based on optofluidic technology. The remainder of the paper is structured as follows: Section 2 introduces the fundamental principles of optofluidic technology and outlines typical fabrication processes. Section 3 reviews recent sensor designs and their applications in measuring key ocean chemical parameters. A comparative analysis of different technologies is presented in Section 4. Section 5 discusses the existing challenges and future development prospects of miniaturized ocean sensors utilizing microfluidic and optofluidic approaches. Finally, Section 6 concludes the paper.

## 2. Optofluidic Technology and Its Fabrication

### 2.1. Optofluidic Technology

Optofluidic is an emerging interdisciplinary field that integrates microfluidic technology with optical detection, enabling highly sensitive analysis of optical signals through the manipulation of liquids within micrometer-scale channels [40]. It is widely utilized in lab-on-chip (LOC) platforms for compact, integrated chemical and biological analysis and measurement [41].

Optofluidic technology is particularly suitable for marine detection due to its ability to integrate microfluidic sample handling with highly sensitive optical measurement in a compact, low-power platform. The microscale channels enable rapid mixing and short diffusion distances, allowing for fast and in situ analysis of chemical parameters with minimal reagent consumption. Additionally, the integration of optical components directly into the fluidic system enhances detection sensitivity and selectivity, which is critical for monitoring low-concentration nutrients or pollutants in oligotrophic ocean regions. These features make optofluidic systems ideal for autonomous, long-term, and real-time marine monitoring. Hence, optofluidic can be an excellent option for miniaturizing ocean parameter sensors [29,42,43]. In the context of marine sensing, optofluidic systems provide a miniaturized and efficient solution for real-time, in situ monitoring of key seawater parameters such as physical parameters, pH, nutrients, dissolved oxygen (DO) and heavy metal ions [44,45,46,47].

### 2.2. Fabrication Techniques

The fabrication of optofluidic sensors employs a range of advanced techniques. Each of them is tailored to specific materials, device architectures and application. This section provides a detailed overview of several key manufacturing methods.

#### 2.2.1. Soft Lithography and Molding Process

Soft lithography is a cost-effective and widely adopted technique for fabricating microfluidic and optofluidic devices, especially those made from elastomeric materials. Soft lithography is considered a flexible alternative to conventional photolithography. It facilitates rapid prototyping and scalable production without the need for expensive cleanroom facilities [48,49].

The fabrication process typically begins with the creation of a rigid master mold. SU-8 photoresist is often used for mold in standard photolithographic methods [50]. This master mold serves as a reusable template for replicating optofluidic systems. Polydimethylsiloxane (PDMS)—the most commonly used elastomer due to its favorable properties such as optical transparency, elasticity and compatibility with rapid prototyping techniques—is poured over the SU-8 mold. After thermal curing, the PDMS layer is carefully peeled off, reproducing a high-fidelity negative replica of the original microstructure [51].

**Figure 1:**
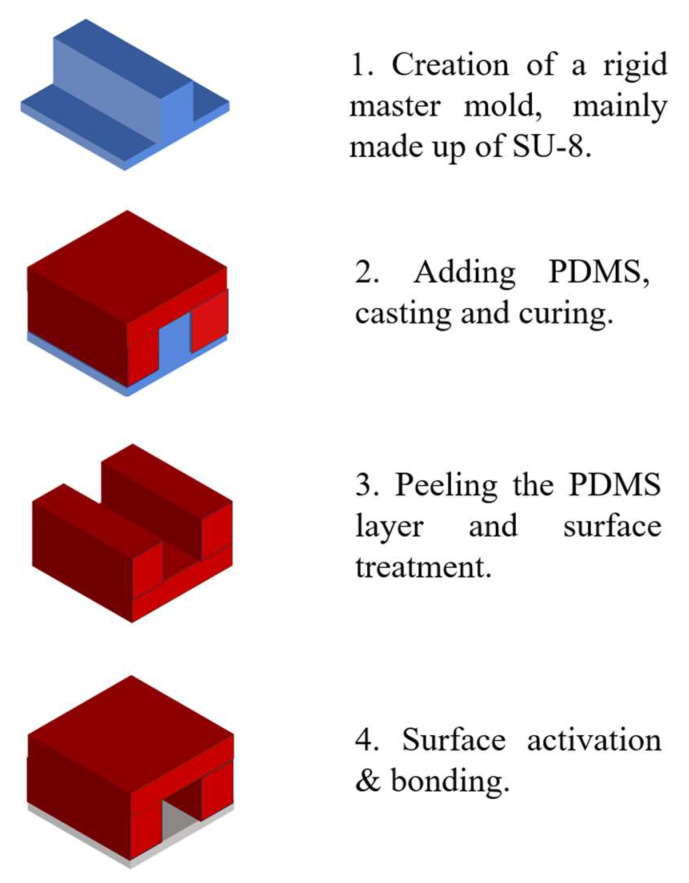
The process of soft lithography.

The patterned PDMS replica, which contains microchannels or embedded optical features, is then sealed onto a substrate. Common substrate materials include glass, silicon, polystyrene, polyethylene, silicon nitride, or an additional PDMS layer. Precise alignment and bonding are critical to prevent fluid leakage and maintain optical accuracy. Plasma bonding—typically using air or oxygen plasma—is the most prevalent method for creating irreversible seals. Plasma treatment generates silanol groups (–SiOH) on the PDMS surface, which react with hydroxyl groups on the substrate to form covalent bonds. Alternatively, irreversible bonding can also be achieved by applying an excess of PDMS prepolymer (monomer and curing agent) to each surface and thermally curing the assembled structure [52]. Figure 1 illustrates the typical soft lithography process used in the fabrication of optofluidic systems.

A variety of materials are employed in the construction of optofluidic devices. PDMS is preferred for microchannel fabrication due to its optical transparency, electrical insulation, low cost [53]. SU-8 photoresist remains the material of choice for creating high-resolution master molds [50]. Substrate selection—ranging from glass and silicon to thermoplastics (e.g., polystyrene, polyethylene) and silicon nitride—depends on the specific optical, chemical, and mechanical requirements of the application.

#### 2.2.2. Femtosecond Laser Process

Femtosecond laser processing is a powerful, maskless, and true three-dimensional (3D) microfabrication technique that utilizes ultrashort laser pulses to directly modify transparent materials. By tightly focusing femtosecond laser pulses, multiphoton absorption is induced within the material, resulting in permanent, spatially localized changes in the refractive index. Because most of the laser energy is absorbed by free electrons before thermal diffusion can occur, the process creates minimal heat-affected zones (HAZ), enabling microscale fabrication with exceptionally high precision [54].

A unique advantage of femtosecond laser processing is its ability to perform internal 3D structuring. By scanning the laser focus in the X–Y–Z directions or moving the sample, arbitrary three-dimensional architectures can be directly inscribed beneath the surface of the material without causing surface damage [55].

For the fabrication of hollow microchannels, femtosecond laser processing is typically combined with a two-step approach: laser writing followed by chemical wet etching. During the laser writing step, femtosecond pulses modify the chemical structure of the irradiated regions, creating a “latent image.” In photosensitive glasses such as Foturan, this process involves the generation of free electrons that reduce silver ions to form silver nanoclusters. Upon thermal treatment, these clusters serve as nucleation sites for the formation of a crystalline lithium metasilicate phase, which is selectively dissolvable in hydrofluoric acid (HF). In fused silica, structural modifications such as a reduction in Si–O–Si bond angles occur, and linearly polarized lasers can induce the formation of periodic nanogratings, which enhance the selectivity of the subsequent etching process [56].

**Figure 2:**
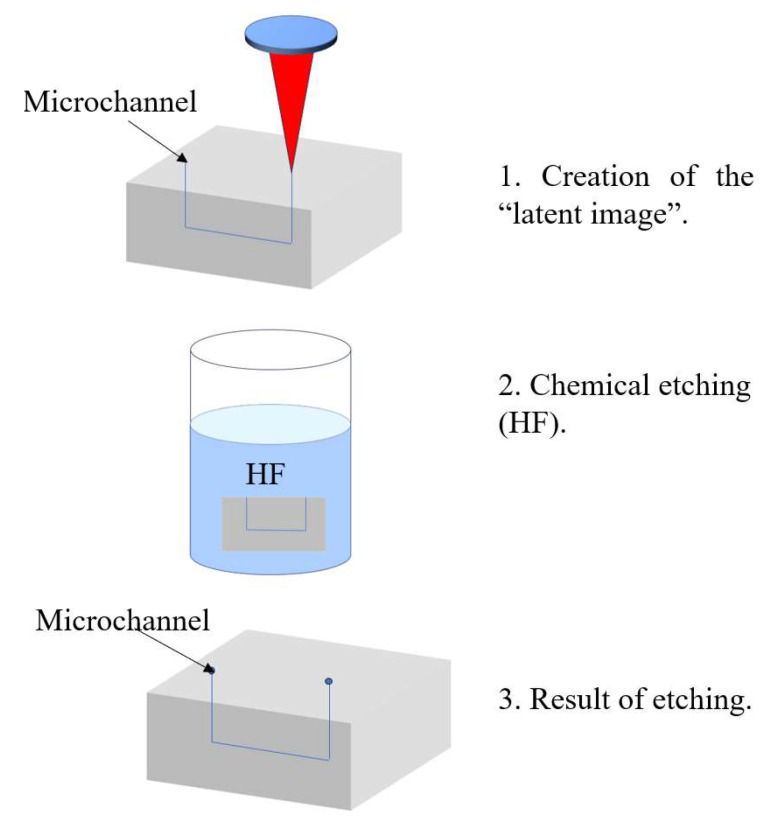
Femtosecond laser process.

The chemically modified regions are then selectively removed using wet chemical etching—typically in an ultrasonic bath with an acidic solution such as HF. The etching rate in laser-modified zones can be up to 50 times higher than in unmodified material, as demonstrated in materials like Foturan [56]. This process enables the fabrication of precise internal microchannels for microfluidic and optofluidic devices. Figure 2 below illustrates the femtosecond laser-assisted fabrication process for optofluidic systems.

Femtosecond laser processing offers several notable advantages: it supports maskless, true 3D patterning with subsurface structuring capabilities; it is compatible with a broad range of materials—including glasses, crystals, and polymers; and it typically requires shorter fabrication times compared to conventional methods. However, the technique also presents some challenges. It demands extremely high-precision laser focusing and motion control, which contributes to its relatively high cost and lower yield rate [57].

#### 2.2.3. 3D-Printing Process

3D printing, specifically stereolithography (SLA), has revolutionized the fabrication of microfluidic and optofluidic devices by enabling additive, layer-by-layer construction. This cutting-edge technique has ushered in a new era of rapid prototyping, miniaturization, and functional integration, allowing for the creation of complex microfluidic and optical architectures essential for reliable in situ sensing in harsh, deep-sea marine environments [58].

SLA uses a focused light source, typically a UV laser, to selectively cure photosensitive resins in successive layers. The typical fabrication process proceeds as follows. First, a 3D model of the sensor—incorporating features such as microfluidic channels and optical cavities—is designed using CAD software. The model is then exported as an STL or OBJ file and sliced into 2D layers. The build platform is immersed in a vat of liquid resin, and a UV laser selectively cures each layer based on the sliced design. Common SLA resins include acrylic resins (e.g., HDDA), polyethylene glycol diacrylate (PEG-DA), polymethyl methacrylate (PMMA), and poly(D,L-lactide) along with their copolymers.

After each layer is cured, the build platform incrementally rises to allow the next layer to form. In some advanced systems, oxygen-permeable windows are used to prevent resin adhesion and accelerate polymerization. Once printing is complete, the structure is rinsed with isopropyl alcohol to remove residual uncured resin. Support structures are removed, and post-processing steps such as UV/thermal curing, surface smoothing, or coatings (e.g., metal, ceramic, or hydrophobic layers) are applied to improve mechanical strength, optical clarity, and corrosion resistance.

**Figure 3:**
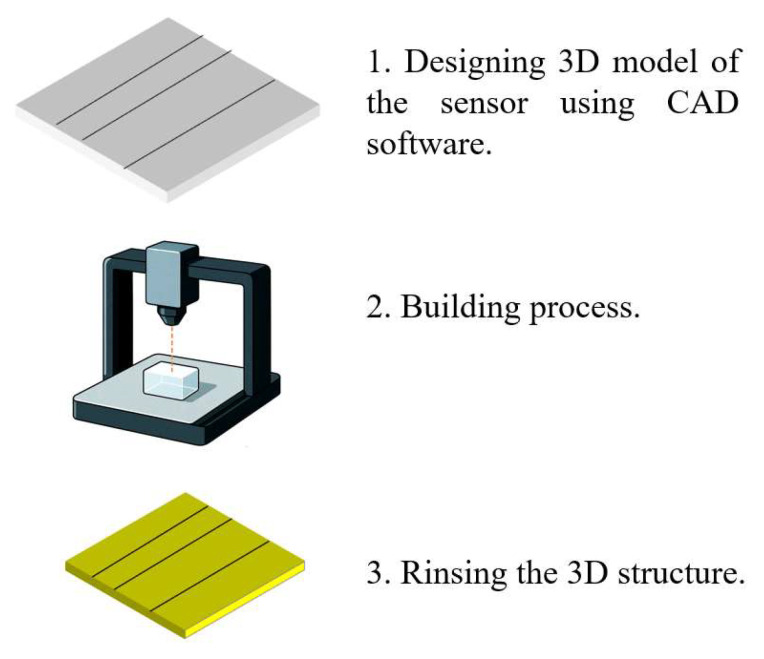
The process of 3D printing.

SLA is particularly well suited for prototyping optofluidic sensor cavities and microchannels, making it an ideal approach for marine environmental sensing applications. Figure 3 illustrates the process of 3D printing for fabricating an optofluidic system.

#### 2.2.4. Comparison Between Three Fabrication Methods

Table 1 below shows the comparison between these three fabrication methods.

## 3. Measurement of Ocean Parameters

There are many essential ocean environmental parameters, including temperature, salinity, pressure, dissolved oxygen (DO), pH, nutrients, and heavy metal ions, which are critical for understanding marine ecosystem dynamics and assessing environmental health. Among these, chemical parameters such as nutrients, pH, DO, and heavy metal ions play a pivotal role in biogeochemical processes and pollution monitoring. This section focuses on the application of optofluidic technology for the measurement of these chemical parameters in marine environments.

### 3.1. Nutrients

Measuring nutrient concentrations in the ocean—particularly nitrate (NO_3_^−^) and phosphate (PO_4_^3−^)—is essential for understanding marine biogeochemical cycles and ecosystem dynamics. These nutrients are fundamental to phytoplankton growth, which lies at the base of the marine food web by fixing carbon dioxide through photosynthesis and supplying energy to higher trophic levels such as fish. Furthermore, anthropogenic nutrient inputs from agriculture and wastewater discharge can lead to coastal eutrophication and the formation of hypoxic zones. Hence, nutrients monitoring is crucial for assessing human impacts on marine ecosystems [59]. Nutrient distribution also influences the spatial patterns and productivity of fisheries, particularly in upwelling regions and nutrient-rich coastal waters. Accurate nutrient measurement, therefore, supports better assessments of primary productivity, ecosystem health, and fisheries management.

Traditionally, marine nutrient sensors have employed reagent-based colorimetric assays, such as cadmium reduction, for nitrate and molybdenum blue for phosphate. These methods remain the gold standard due to their chemical specificity and robustness. Recently, microfluidic lab-on-chip (LOC) platforms have enabled the miniaturization and automation of these colorimetric reactions, significantly reducing reagent consumption, analysis time, and overall system footprint—key advantages for long-term in situ ocean deployments [60].

In colorimetric analysis systems, chemical reagents are introduced into microchannels where they react with target analytes to produce a color change [61]. This colorimetric shift is quantitatively analyzed by measuring the variation in light intensity according to the Beer–Lambert law, which relates absorbance to the concentration of the absorbing species [62]:

(1) $$\log\frac{I_{0}}{I}=A=\varepsilon lc$$

Here, *A* is the absorbance, *ε* is the molar absorptivity, *c* is the concentration, and *l* is the optical path length. In optofluidic systems, the microchannel itself acts as the optical path, while integrated optical components—typically LEDs and photodetectors—measure absorbance at specific wavelengths. This configuration enables high sensitivity and specificity even with small sample volumes, making it particularly suitable for marine applications where nutrient concentrations are often low.

#### 3.1.1. Phosphate

Generally, two common spectrophotometric methods are used for phosphate determination: the molybdenum blue and molybdenum yellow methods. In the molybdenum blue method, under acidic conditions, phosphate reacts with ammonium molybdate to form phosphomolybdic acid, which is then reduced by a reducing agent to yield a blue complex. This complex exhibits strong absorbance at around 880 nm and offers high sensitivity. This feature makes it particularly suitable for trace-level analysis [63]. In contrast, an unreduced yellow complex is formed in the molybdenum yellow method, with an absorption peak between 340 and 400 nm [64]. It is simpler to operate and well-suited for rapid detection and automated analysis, but its sensitivity is relatively lower. Consequently, the molybdenum blue method is more widely adopted when higher precision is required. Table 2 summarizes the comparison between the two methods.

In 2017, Zhu et al. developed a phosphate detection system integrated with a Fabry–Perot (F–P) resonator [67]. This optofluidic platform achieved a limit of detection (LOD) of 0.1 μmol/L, a detection range of 0–100 μmol/L, a response time of 6 s, and reagent consumption of just 6 μL [67]. In this system, chromogenic agent A consists of an ascorbic acid solution, while chromogenic agent B comprises a mixture of 12% ammonium molybdate, 80% concentrated sulfuric acid, and 8% antimony potassium tartrate. The Fabry–Pérot resonator was employed to enhance detection precision by extending the optical path length.

In 2022, Morgan et al. proposed an in situ submersible phosphate analyzer deployed in the Stella Maris testbed, located approximately 100 m offshore and 9 m deep at the inlet to the Bedford Basin in Nova Scotia, Canada [68]. The system successfully completed over 300 measurements of phosphate, achieving an LOD of 0.0152 μmol/L, a limit of quantification of 0.0508 μmol/L, and a detection range of 0.2–10 μmol/L [68].

In 2024, Zhao et al. demonstrated a microfluidic phosphate analyzer based on liquid waveguide capillary cells (LWCC), marking the first use of LWCCs in microfluidic devices for seawater phosphate detection [47]. This design achieved an impressive LOD of 8 nmol/L and a detection range of 0–23 μmol/L. The system has been deployed in the Pearl River Estuary, Daya Bay, and used for sampling in the Xisha Sea area. This sensor shows high reliability for in situ nutrient monitoring [47].

#### 3.1.2. Nitrate and Nitrite

Spectroscopic detection, electrochemical detection, chromatography and capillary electrophoresis are commonly employed for the measurement of nitrate and nitrite in aquatic environments [69]. Among these, spectrophotometric methods are widely favored due to their simplicity, low cost and high sensitivity. Nitrite (NO_2_^−^) can be directly quantified using the Griess reaction [70]. In this action, sulfanilamide reacts with nitrite under acidic conditions and form a diazonium salt. It subsequently couples with N-(1-naphthyl) ethylenediamine to produce a pink azo dye, measurable at 540 nm. Nitrate (NO_3_^−^) determination requires a reduction step to convert nitrate into nitrite. This reduction can be achieved using cadmium–copper columns, vanadium(III) chloride (VCl_3_), or other chemical reductants [71,72]. The resulting nitrite is then quantified using the same Griess colorimetric approach. Modern systems, such as automated or flow-injection platforms, are capable of sequentially detecting NO_2_^−^ and NO_3_^−^ in a single run with high efficiency.

In 2015, Cogan et al. developed a low-cost and stable optofluidic sensor for nitrate detection, marking the first application of chromotropic acid in an optofluidic system [73]. In this design, nitrate reacts to produce a yellow-colored complex with strong absorption at 430 nm, achieving LOD of 0.31 mg/L and a detection range of 0–80 mg/L [73].

In 2019, Nightingale’s team introduced a droplet-based microfluidic nitrate/nitrite sensor capable of ultra-high-frequency sampling—achieving one measurement every 10 s for a three-week deployment in the Itchen river basin (UK)—while consuming only 2.8 mL of reagent every day [74]. Compared to traditional continuous flow injection analysis, this system significantly reduces reagent consumption and increases sampling efficiency [74].

Beaton et al. later showed a multi-channel LOC nutrient analyzer capable of full-spectrum detection of both nitrate and phosphate. Deployed in deep-sea environments at depths of up to 4800 m, this system achieved average LODs of approximately 0.03 μmol/L for nitrate and 0.016 μmol/L for phosphate [75]. The device was successfully integrated into CTD rosettes and profiling floats, enabling vertical profiling of nutrient concentrations throughout the water column of the Mediterranean Sea [75].

#### 3.1.3. Comparison Between Traditional Methods and Optofluidic Technology

Optofluidic technology and traditional methods each have their own advantages and disadvantages in detecting marine nutrients. Table 3 compares optofluidic technology with traditional methods.

### 3.2. pH

Accurate pH measurement is a critical aspect of oceanographic studies [86,87,88]. The ocean plays a vital role in the global carbon cycle by absorbing approximately one-quarter of anthropogenic CO_2_ emissions. This absorption leads to the formation of carbonic acid in seawater, which lowers its pH—a process known as ocean acidification. Ocean acidification poses significant threats to marine ecosystems, particularly to calcifying organisms such as corals and shellfish that rely on stable carbonate chemistry. Therefore, the development of pH sensors capable of in situ, long-term, miniaturized, and precise measurements is essential for effective ocean monitoring systems [87].

To measure pH accurately, it is important first to clarify its definition. For simple aqueous solutions, pH is defined as the negative base-10 logarithm of the hydrogen ion activity:

(2) $$pH=-\log\left(a_{H^{+}}\right)$$

When the solution is sufficiently diluted and the ionic strength approaches zero, *a_H_*^+^ = [H^+^]. However, seawater is a complex solution with high ionic strength, where the activity of hydrogen ions deviates significantly from their concentration due to electrostatic shielding and extensive ion–ion interactions. To address these non-ideal effects and ensure consistency in describing chemical equilibria, different pH scales have been established for seawater: the free scale (*pH_F_*), total scale (*pH_T_*), and seawater scale (*pH_SWS_*) [88].

The free scale (*pH_F_*) represents the fundamental pH definition based solely on the concentration of free hydrogen ions, excluding ion pairs or complexes:

(3) $$pH_{F}=-\log{\left[H^{+}\right]}_{free}$$

It is widely used in theoretical chemistry and modeling but tends to underestimate acidity in seawater due to ignoring ion interactions.

The total scale (*pH_T_*) incorporates both free hydrogen ions and bisulfate ions (HSO_4_^−^), formed by the interaction of H^+^ with sulfate ions (SO_4_^2−^):

(4) $$pH_{T}=-\log\left({\left[H^{+}\right]}_{free}+\left[HSO_{4}^{-}\right]\right)$$

This scale is among the most commonly used in oceanography, particularly for spectrophotometric pH measurements utilizing indicators such as m-cresol purple (mCP).

The seawater scale (*pH_SWS_*) further adds the contribution of fluoride ion (HF) along with bisulfate:

(5) $$pH_{SWS}=-\log\left({\left[H^{+}\right]}_{free}+\left[HSO_{4}^{-}\right]+\left[HF\right]\right)$$

This scale is used in advanced modeling and thermodynamic studies but is less common in routine measurements due to its complexity. Table 4 summarizes the differences between these scales.

For high-precision marine pH measurements, m-cresol purple (mCP) is a widely used colorimetric indicator. It exhibits distinct color changes at different pH values, with characteristic absorbance peaks near 434 nm and 578 nm, enabling accurate spectrophotometric determination of pH [89]. The seawater pH measurement using mCP follows the equation [89]:

(6) $$pH_{T}=-\log\left(K_{2}^{T}\right)+\log\left(\frac{R-e_{1}}{e_{2}-Re_{3}}\right)$$

where *K*_2_*^T^* is the dissociation constant of the indicator, *R* = *A*_578_/*A*_434_ is the absorbance ratio, and *e*_1_, *e*_2_ and *e*_3_ are ratios of molar extinction coefficients of mCP:

(7) $$e_{1}=\frac{\varepsilon_{578}^{HI}}{\varepsilon_{434}^{HI}},\;e_{2}=\frac{\varepsilon_{578}^{I}}{\varepsilon_{434}^{HI}},\;e_{3}=\frac{\varepsilon_{434}^{I}}{\varepsilon_{434}^{HI}}$$

However, this equation has limitations in precision. In 2011, Liu et al. proposed an improved formula for measuring seawater pH with pure mCP [90]:

(8) $$pH_{T}=-\log\left(K_{2}^{T}e_{2}\right)+\log\left(\frac{R-e_{1}}{1-R\frac{e_{3}}{e_{2}}}\right)$$

This approach enhances measurement precision, reducing pH error from 0.01–0.02 to approximately 0.005 [91]. Because mCP is sensitive to salinity and temperature variations in seawater, calibration of its molar extinction coefficients and dissociation constants is necessary for accurate measurement [45]. Furthermore, the pH change caused by adding mCP to seawater cannot be neglected in high-precision applications. Under high-pressure conditions, chemical properties of mCP may also change, affecting pK_a_ and extinction coefficients.

In 2021, Yin et al. reported a LOC pH sensor based on spectroscopy capable of operating at depths up to 6000 m [92]. This system mixes seawater samples with indicators on-chip and measures pH via colorimetry, achieving high accuracy (<0.001), low power consumption (3 W), and minimal reagent usage (3 μL per measurement). Field tests showed the device maintains a pH measurement error between approximately 0.003 and 0.022 [92].

Both optofluidic technology and traditional methods for marine pH detection offer distinct advantages and limitations. Table 5 compares optofluidic technology with traditional methods.

### 3.3. Dissolved Oxygen (DO)

DO is a key indicator of marine water quality, reflecting both ecological stability and environmental health. It directly influences the survival, growth, and reproduction of marine organisms, making it essential for maintaining balanced ocean ecosystems [93]. Variations in DO levels often signal pollution or eutrophication events and serve as early warnings of hypoxic or “dead zones” that can lead to large-scale marine die-offs. In fisheries management, DO concentration plays a critical role in habitat suitability assessments, as many fish species exhibit strong behavioral and distributional responses to oxygen availability [94]. Furthermore, in coastal engineering and development projects, real-time DO monitoring is vital for evaluating environmental impact and project feasibility. Consequently, the development of high-precision, real-time, and miniaturized DO sensors is essential for preserving marine ecosystem health, supporting sustainable resource utilization, and advancing global climate research.

Optofluidic technology presents a promising solution for the development of compact, low-power DO sensors by combining microfluidics with integrated optical detection. A commonly employed principle is fluorescence (or phosphorescence) quenching, wherein oxygen-sensitive dyes—typically platinum(II), ruthenium(II), or iridium complexes—are immobilized within microfluidic channels. Upon excitation, usually by a light-emitting diode (LED), the luminescence of these dyes is quenched in the presence of oxygen, resulting in measurable reductions in intensity or fluorescence lifetime [95]. This optical response is captured using integrated photodetectors, waveguides, or optical fibers. The quantitative relationship between the luminescence signal and oxygen concentration is described by the Stern–Volmer equation [96]:

(9) $$\frac{I_{0}}{I}=\frac{\tau_{0}}{\tau }=1+K_{SV}\left[Q\right]$$

Here, *I_0_* and *I* denote the fluorescence intensity in the absence and presence of DO, respectively; *τ_0_* and *τ* are the corresponding lifetimes; *K_SV_* is the Stern–Volmer constant, reflecting the quenching efficiency; and [*Q*] represents DO concentration.

In 2019, a notable study demonstrated an optofluidic DO sensor utilizing fluorescence quenching enhanced by total internal reflection within a multilayer microchannel, achieving significantly improved sensitivity compared to traditional designs [97]. In 2021, Wang et al. developed a PDMS-based chip coated with a platinum(II) meso-tetrakis (pentafluorophenyl) porphyrin (PtTFPP) sensing membrane. This sensor demonstrated a fast response time of 22 s and a LOD of 0.01 mg/L DO [98].

Compared to conventional Clark-type amperometric sensors, optofluidic DO sensors offer significant advantages in terms of sensitivity, stability, and operational autonomy. The Clark sensor is an electrochemical device to measure DO in liquids. It consists of a platinum cathode and a silver/silver chloride (Ag/AgCl) anode, separated from the sample solution by an oxygen-permeable membrane. When a constant voltage is applied, oxygen diffuses through the membrane and is reduced at the cathode. This results in a current proportional to the oxygen concentration [99]. However, problems, like membrane diffusion delays, susceptibility to fouling and electrolyte degradation, limit their response speed and long-term stability [100].

In contrast, optofluidic DO sensors offer faster and more sensitive optical responses, consume no DO in the detection process, and require less maintenance. Their resistance to flow rate and temperature fluctuations, along with their compatibility with autonomous and in situ deployment, make them especially suitable for long-term ocean monitoring [100].

### 3.4. Heavy Metal Ion

Heavy metals such as lead (Pb), mercury (Hg), arsenic (As), cadmium (Cd), and chromium (Cr) are pervasive aquatic pollutants that bioaccumulate in organisms and pose significant ecological and health hazards.

Conventional detection methods for heavy metal ions in marine environments—such as atomic absorption spectroscopy (AAS), inductively coupled plasma mass spectrometry (ICP-MS), and electrochemical techniques like anodic stripping voltammetry (ASV)—offer high accuracy and ultra-low detection limits. However, these techniques typically require bulky, expensive instruments, skilled personnel, and extensive sample preparation, making them unsuitable for real-time, in situ monitoring.

In contrast, optofluidic platforms, which integrate microfluidic handling with optical detection methods (e.g., colorimetric and fluorescence assays), enable the development of compact, low-cost LOC devices. These systems feature rapid response, minimal sample handling, and the potential for autonomous deployment. Despite ongoing challenges such as multi-ion detection, long-term operational stability, and resistance to harsh marine conditions, optofluidic technologies offer comparable sensitivity and high integration, making them especially promising for field and oceanographic applications [101,102].

#### 3.4.1. Absorbance-Based Method

Absorbance-based microfluidic systems have emerged as powerful tools for heavy metal quantification in marine settings by combining the precision of colorimetric assays with the portability and efficiency of LOC platforms [103]. The fundamental principle relies on the formation of a colored complex between heavy metal ions and specific reagents. When light of a specific wavelength passes through the solution, the complex absorbs light, leading to a reduction in transmitted intensity. The concentration of metal ions is determined by measuring this absorbance change. For instance, lead ion (Pb^2+^) detection can be achieved using AB/PPD-1 as a colorimetric reagent, which forms a complex (Pb(II)-AB/PPD-1) exhibiting a characteristic absorbance peak near 490 nm [46].

In 2015, Milani et al. developed a microfluidic in situ colorimetric analyzer for iron (Fe) and manganese (Mn) detection, achieving low-cost implementation with detection limits of 27 nmol/L and 28 nmol/L for Fe and Mn, respectively [104]. And it was deployed over 16 days in the Baltic Proper, attaching to the IOW-Pump–CTD system. Its stability was demonstrated through multiple successful deployments at depths exceeding 300 m, even under challenging weather conditions.

In 2019, Lace et al. reported an optofluidic system for arsenic (As) detection based on the leucomalachite green method [105]. Arsenic reacts with potassium iodate to generate iodine, which converts leucomalachite green into malachite green with an absorbance peak at 617 nm. The system achieved a detection limit of 0.32 mg/L with a linear range of 0.3–2 mg/L [105].

#### 3.4.2. Fluorescence-Based Method

Fluorescence-based methods—including those using fluorescent aptamers, quantum dots (QDs), and organic dyes—offer high sensitivity and low detection limits [106].

Fluorescence quenching and aptamer-based strategies provide distinct approaches for the detection of heavy metal ion in marine environments. Fluorescence quenching methods use the ability of metal ions to diminish fluorophore emission via processes such as electron or energy transfer, including Förster resonance energy transfer (FRET). These methods are straightforward and rapid, but their practical application in seawater is often limited by insufficient selectivity and strong interference from coexisting ions and organic matter.

**Figure 4:**
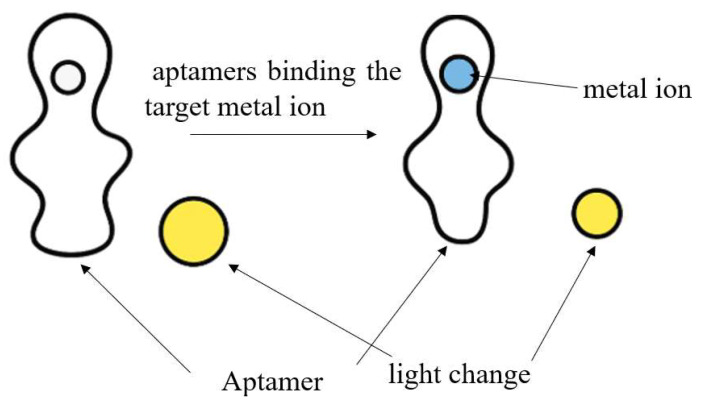
The process of the heavy metal ion sequestered by the aptamer.

By contrast, aptamer-based assays employ nucleic acid ligands with high affinity and specificity toward target metal ions. Fluorescent aptamer sensors, for instance, leverage the high specificity of nucleic acid aptamers and the sensitivity of fluorescence detection. For Hg^2+^, Pb^2+^, and Cd^2+^, aptamers bind the target ion (e.g., via T–Hg^2+^–T base pairing or G-quadruplex structures), resulting in structural changes which can be transduced into detectable fluorescence changes, frequently through FRET-based readout. Despite the higher cost and design complexity, aptamer-based methods offer superior selectivity, enhanced sensitivity, and improved adaptability for trace analysis in complex marine environment [106,107]. Figure 4 below shows how heavy metal ion is sequestered by the aptamer.

In 2019, Park and Seo designed an integrated optofluidic sensor combining solid-phase extraction and graphene oxide quantum dots to detect As^3+^, Cd^2+^, and Pb^2+^, achieving LODs of 5.03 nmol/L, 41.1 nmol/L, and 4.44 nmol/L, respectively [108].

Zhou et al., in 2020, introduced a paper-based 3D rotary microfluidic chip utilizing ZnSe QDs and ion imprinting for simultaneous detection of Cd^2+^ and Pb^2+^, achieving LODs of 0.245 μg/L and 0.335 μg/L, with linear ranges of 1–70 μg/L and 1–60 μg/L, respectively [109].

In 2021, Huang et al. developed an aptamer-based optofluidic sensor using graphene oxide as a quencher to measure Hg^2+^ and Pb^2+^, with LODs of 0.70 ppb and 0.53 ppb, respectively [110]. In these systems, aptamers labeled with fluorophores such as FAM or HEX are quenched by graphene oxide (GO) via FRET. Upon binding target ions, aptamer conformation changes reduce FRET, restoring fluorescence.

In 2024, Li et al. demonstrated a microfluidic fluorescent sensor array using molecular probes to simultaneously detect Hg^2+^, Pb^2+^, Cr^3+^, and Cu^2+^, achieving LODs of 0.89 nmol/L, 9.60 nmol/L, 5.45 nmol/L, and 1.77 nmol/L, respectively [111].

## 4. Comparison Between Optofluidic and Traditional Sensors

The table below compares traditional MEMS-based sensors with microfluidic and optofluidic platforms across key performance metrics. In general, lab-on-chip (LOC) devices offer enhanced sensitivity and require significantly lower reagent and sample volumes compared to conventional bulk sensors, while also supporting multifunctional integration. Optofluidic platforms further incorporate optical interrogation into microfluidic systems, often achieving superior sensitivity and enabling real-time, high-resolution detection. summarizes the performance comparison among these different sensing technologies. Table 6 summarizes the performance comparison among these different sensing technologies.

## 5. Challenges and Prospects

**Figure 5:**
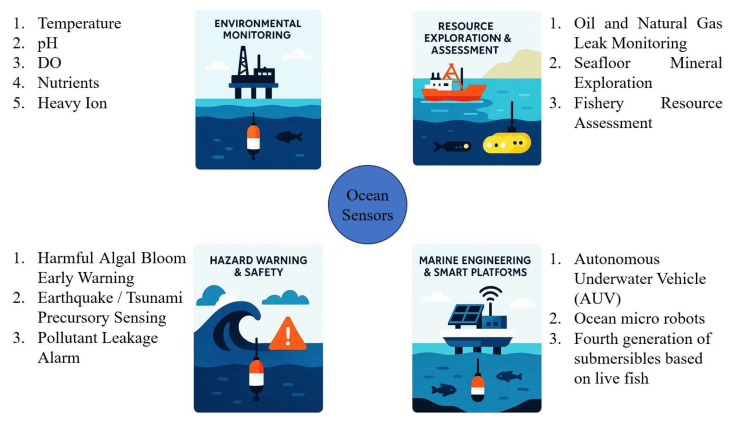
The application of ocean sensors.

Miniaturized ocean sensors have a wide variety of applications. Figure 5 shows some applications of ocean sensors.

The sensors and detectors based on optofluidic technology offer many advantages over traditional electronic-based systems, including high sensitivity, compact size, resistance to electromagnetic interference, corrosion resistance to seawater, and capability for remote sensing. Compared to conventional nutrient, pH, dissolved oxygen (DO) and heavy metal ion analyzers, optofluidic sensors offer significant advantages in miniaturization, low sample consumption, fast response, and potential for integration. The use of microfluidic channels enables efficient reagent mixing and rapid optical detection in compact platforms. These make them highly suitable for autonomous, in situ marine nutrient monitoring, particularly in challenging deep-sea environments where size, power, and maintenance constraints are critical.

Nonetheless, despite these advantages, the development and deployment of miniaturized ocean sensors still face several technical and practical challenges that must be addressed to enable widespread application.

### 5.1. Endurance of Sensors

For long-duration remote voyages and deep-sea exploration, battery life is a critical factor influencing the performance and deployment duration of ocean sensors. Future improvements in endurance are expected to focus on two key strategies: enhancing battery energy density and minimizing power consumption.

In optofluidic sensors—which integrate microfluidic channels with optical detection components—power consumption primarily arises from light sources, micro-pumps, and control electronics. Several approaches can improve energy efficiency without compromising performance, even within the constraints of miniaturized systems.

First, low-power pulsed light-emitting diodes (LEDs) can be employed in place of continuous illumination, substantially reducing optical energy requirements while maintaining adequate signal quality. Second, passive microfluidic actuation methods, such as capillary-driven flow, offer energy-efficient alternatives to traditional active micro-pumps, which typically consume substantial power [74,113,114]. Third, the integration of ultra-low-power system-on-chip (SoC) solutions for signal processing and control enables significantly lower standby and active power consumption, improving overall system efficiency. Additionally, intermittent or event-triggered data transmission strategies can further reduce energy demands. In such systems, data are transmitted at predefined intervals or only when critical events are detected, instead of continuously. During inactive periods, both the sensing elements and communication modules enter low-power sleep modes, potentially reducing energy usage by an order of magnitude [115].

### 5.2. Breakthroughs in Materials and Packaging Technologies for Ocean Sensors

The future development of ocean optofluidic sensors will be closely linked to ongoing advancements in materials science and packaging technologies. As ocean monitoring increasingly demands longer deployment durations, higher precision, and seamless integration into autonomous platforms, future sensor systems must become more robust, intelligent, and miniaturized.

One key direction lies in the development of smart, self-healing, and biofouling-resistant materials. Biofouling remains a major obstacle in long-term marine sensing, often compromising optical performance and necessitating frequent maintenance. Next-generation sensor surfaces will leverage stimuli-responsive polymers that modify surface energy or release antifouling agents in response to environmental cues such as pH, light, or salinity, effectively mitigating the accumulation of microorganisms and debris [112]. Recent reviews have emphasized non-toxic, environmentally friendly nanocoatings that offer biocide-free fouling resistance and demonstrate strong durability in marine environments [116,117]. Additionally, shark-skin-inspired surface structures have been shown to reduce microfouling settlement by approximately 77% [118], offering a promising bioinspired approach.

Environmental sustainability is also a critical consideration. As the use of single-use sensors and swarm-based platforms expands, biodegradable packaging becomes essential to minimize long-term ecological impact. Studies have demonstrated the feasibility of using biodegradable polymers—such as polylactic acid (PLA) and polyhydroxyalkanoates (PHAs)—which provide sufficient mechanical strength during deployment but degrade naturally over time [119,120]. Future research must focus on optimizing material formulations to ensure complete degradation without the release of microplastics, thereby ensuring true environmental compatibility.

### 5.3. Data Transfer and Processing Technology

As demand increases for continuous, autonomous, and intelligent ocean monitoring, optofluidic sensors will increasingly depend on advances in on-sensor artificial intelligence (AI), hybrid communication systems, energy-efficient architectures, and seamless edge-to-cloud integration to address emerging challenges.

The integration of compact neural networks into sensor nodes will enable real-time anomaly detection, on-site calibration updates, and adaptive data acquisition, thereby reducing bandwidth requirements and enhancing operational autonomy [121]. Future generations of ocean optofluidic sensors are expected to incorporate neuromorphic processors or ultra-low-power AI accelerators, enabling localized data processing, intelligent calibration, and event-driven sensing—all while operating within tight power and size constraints [122].

In terms of communication, underwater acoustic wireless communication (UAWC) is currently the most widely used approach due to its long transmission range—extending several kilometers or more. However, it suffers from low data rates (typically in the kilobits per second range) and is highly susceptible to environmental factors such as noise, temperature gradients, and ocean currents, which lead to signal attenuation and transmission delays. In contrast, underwater optical wireless communication (UOWC) offers exceptionally high data rates (up to megabits or even gigabits per second), making it ideal for short-range, high-bandwidth applications. However, its effective transmission distance is typically limited to tens to hundreds of meters [123,124]. To leverage the strengths of both modalities, hybrid optical–acoustic wireless communication systems are envisioned for future optofluidic platforms. These systems would combine the long-range capability of UAWC with the high-speed transmission of UOWC, enabling both efficient long-distance data relay and high-bandwidth local communication. Such hybrid architectures will be essential for scalable, flexible, and real-time ocean sensor networks in increasingly complex marine environments.

### 5.4. Prospects of Micro Robots and Submersibles Based on Live Fish

Optofluidic sensors hold significant potential for integration into underwater robotic systems, which are increasingly central to modern ocean exploration and monitoring. Recent advancements in this field have led to two distinct yet complementary approaches: underwater micro robots and live-fish-based fourth-generation submersibles.

Underwater micro robots, inspired by the locomotion mechanisms of small aquatic organisms, adopt propulsion strategies such as flagellar motion, pulling, and jet propulsion to achieve efficient mobility. In complex and sensitive environments—such as coral reefs, sediment layers, and seabed fissures—swarms of micro-robots can collect key chemical parameters (e.g., pH, heavy metal ions) with high spatial resolution and minimal ecological disturbance. Additionally, these robots can dynamically track and capture marine microorganisms or pollutant particles such as microplastics, supporting both real-time environmental sensing and seawater remediation [125,126].

Despite their promise, several critical challenges persist. Power supply remains a major bottleneck, as micro-robots have limited onboard energy capacity for long-term autonomous operation. Furthermore, underwater communication at small scales is constrained by limited bandwidth and poor signal penetration. Localization also poses difficulties, as traditional GPS does not function underwater, and microscale underwater positioning technologies are still underdeveloped, limiting precise navigation and coordination [127].

In contrast, the fourth generation of submersibles—based on live fish—leverages the biological capabilities of real fish as both actuators and sensing platforms [16]. These systems guide fish using stimuli such as optical, magnetic, or electrical cues, enabling them to follow pre-defined routes [128,129,130]. Live-fish-based submersibles are particularly well-suited for covert operations, including military reconnaissance or infrastructure inspection, as well as ecological monitoring, where onboard or implanted sensors can infer environmental changes based on fish behavior and physiological responses. These systems offer key advantages, including extremely low propulsion noise, minimal energy consumption, and inherent adaptability to dynamic hydrodynamic conditions, such as active obstacle avoidance and environment-driven navigation.

However, this approach also faces substantial challenges. Precisely controlling individual fish behavior is tough because each fish is different and unpredictable. Moreover, attaching sensors or using electrical or chemical methods to influence behavior raises ethical and biosafety concerns. How to ensure long-term biocompatibility of attached hardware without negatively impacting fish health and its natural behavior is also an unsolved issue.

In summary, both micro robots and live-fish-based submersibles represent future directions for ocean exploration and environmental sensing. Their complementary strengths could play a key role in advancing autonomous and minimally invasive ocean monitoring systems.

## 6. Conclusions

Compared with traditional bulk sensors, optofluidic systems offer several key advantages, including miniaturization, high sensitivity, low reagent consumption and real-time measurement capabilities. The chip-based design significantly reduces the overall device size and makes it well-suited for deployment on autonomous platforms such as micro robots and live-fish-based submersibles. High detection precision and low signal-to-noise can be achieved by the combination of microscale control and optical monitor. Additionally, microfluidic operation largely reduces sample and reagent volumes. This lowers both environmental impact and operational costs. These sensors are capable of continuous operation in harsh marine environments without bulky instruments or laboratory-based processing.

In conclusion, this paper has reviewed the current progress in optofluidic ocean sensors and focused on key analytes monitor such as nutrients, pH, dissolved oxygen (DO), and heavy metal ions. It also discusses future directions for improving optofluidic systems in marine applications. While miniaturization based on optofluidic offers transformative potential for marine environmental monitoring, various engineering and scientific challenges also remain to be solved. To realize the full potential of optofluidic sensors requires continued interdisciplinary research combining photonics, materials science, fluid dynamics and data science.

Furthermore, emerging underwater robotic platforms—such as micro-robots and fourth-generation live-fish-based submersibles—demand compact, low-power, and high-performance sensors. Optofluidic technology is expected to play a key role in these next-generation systems. In the long term, optofluidic sensors will become widely deployed tools in ocean exploration and environmental monitoring.

## Figures and Tables

**Table 1:** Comparison between three methods.

Methods	Advantages	Disadvantages
Soft Lithography	Simple and low-cost Biocompatible Suitable for mass replication [50,51,57]	Mold making required Long fabrication time [50,51,57]
Femtosecond Laser	Short fabrication time No mold required Widely for several materials [54,55,56]	Requiring high-precision laser motion control Requiring additional chemical etching [54,55,56]
3D-Print Process	Low-cost and accessible [57,58]	Long fabrication time Limited resolution [57,58]

**Table 2:** Comparison between molybdenum blue and molybdenum yellow analysis.

Properties	Molybdenum Blue	Molybdenum Yellow
Absorption Peak	~880 nm (secondary ~700–720 nm) [63,65]	340–400 nm (often ~400 nm) [64,66]
Sensitivity	High	Moderate
Detection Limit	Relatively Small	Higher (less precise)
Complexity	More steps (reagent reduction)	Simpler, fewer reagents

**Table 3:** Comparison between traditional methods and optofluidic technology for nutrient detection.

Methods	Working Principle	Reagents	Power Consumption	LOD	Response Time	Size
Optofluidic/Lab-on-Chip	Microfluidics and on-chip optics using colorimetry or absorbance	Little	Low (1–2 W; 500–756 J per measurement [60,76])	nmol/L (0.01–0.1 μmol/L [42,67,75])	Fast (s)	Highly miniaturized
UV Spectrophotometry	UV absorption (e.g., nitrate’s absorption peak)	No need	Medium (3–8 W [77,78])	Sub-μmol/L to nmol/L (e.g., approximately 0.2 μmol/L for nitrate [79,80])	Fast (s)	Medium to small
Ion-Selective Electrodes (ISE)	Ion selective membrane pro-duces voltage proportional to analyte activity	No need	Very low (less than 0.1 W)	μmol/L (eg., approximately 0.3–0.7 μmol/L for nitrate [81]; 0.6–1 μmol/L for phosphate [82])	Fast (s)	Small
Auto-Analyzer (Flow Injection)	Miniaturized lab analyzers using pumps and reagent flow	Large	High (about 10 W [83])	μmol/L (approximately 0.4–0.9 μmol/L [84,85])	Slow (min)	Medium to large

**Table 4:** Comparison between different scales.

Scale	Definition	Application	Advantages and Disadvantages
Free scale (pH_F_)	Only free hydrogen ions [H^+^]	Theoretical model	Simple, but ignores ion pairing
Total scale (pH_T_)	[H^+^] and [HSO_4_^−^]	Commonly used model	Accurate and widely used
Seawater scale (pH_SWS_)	[H^+^], [HSO_4_^−^] and [HF]	High-precision model	Most comprehensive, but less commonly used

**Table 5:** Comparison between traditional methods and optofluidic technology.

Methods	Principle	Accuracy	Reagent Use	Power Consumption	Integration and Scalability	Sustainability
Optofluidic LOC Sensor	Microfluidic mixing + color detection	±0.003–0.022 accuracy	Micro-volumes (approximately 3–10 µL per measurement)	Low (~3 W continuous; ~1300 J per measurement)	High integration; miniaturized LOC platform	Good
Glass Electrode	Electrochemical glass membrane	±0.05 pH units	None	Moderate	Bulky; not microfluidic	Requiring frequent maintenance
ISFET Sensor	Field-effect transistor sensitive to H^+^	±0.02–0.05 pH units	None	Low	Medium; can integrate but less proven	Good

**Table 6:** Comparison between different technology.

Properties	Traditional MEMS [112]	Microfluidic [38,57]	Optofluidic [42,57,61]
Detection Method	Electrochemical, capacitive, resistive	Absorbance, electrochemical	Absorbance, fluorescence
Sensitivity	Moderate	High	Very high
LOD	Typically μmol/L	Sub-μmol/L to nmol/L	Nmol/L
Power Consumption	Relatively high	Low	Extremely low (especially for passive microfluidic actuation mechanisms)
Cost of Fabrication	High	Low to moderate	Moderate to high
Advantages	Robust, well-known tech	Reagent efficient, automated	High sensitivity and low LOD
Limitations	Limited multiplexing; higher power	Reagent handling; potential clogging	Complex to fabricate, limited materials, demanding optical design

## Data Availability

Not applicable.
